# Prediction model of gonadotropin starting dose and its clinical application in controlled ovarian stimulation

**DOI:** 10.1186/s12884-022-05152-6

**Published:** 2022-11-04

**Authors:** Liang Hua, Yang Zhe, Yang Jing, Shen Fujin, Chen Jiao, Liu Liu

**Affiliations:** 1 Department of Obstetrics and Gynecology, Renmin Hospital of Wuhan University, Wuhan, China; 2 Reproductive Medicine Center, Renmin Hospital of Wuhan University, Wuhan, China

**Keywords:** Machine learning, Gn starting dose, Controlled ovarian stimulation, In-vitro fertilization

## Abstract

**Background:**

Selecting an appropriate and personalized Gn starting dose (GSD) is an essential procedure for determining the quality and quantity of oocytes in the controlled ovarian stimulation (COS) process of the in-vitro fertilization (IVF) treatment cycle. The current approach for determining the GSD is mainly based on the experience of a clinician, lacking unified and scientific standards. This study aims to establish a prediction model of GSD, based on which good COS outcomes can be achieved with the influencing factors comprehensively evaluated quantitatively.

**Material and methods:**

We collected a total of 1555 patients undergoing the first oocytes retrieving cycle and conducted correlation analysis to find the significant factors related to the GSD. Two GSD models are built based on two popular machine learning approaches, and the one with better model performance is selected as the final model. Finally, clinical application and validation were conducted to verify the effectiveness of the proposed model.

**Results:**

(1) Age, duration of infertility, type of infertility, body mass index (BMI), antral follicle count (AFC), basal follicle stimulating hormone (bFSH), estradiol (E_2_), luteinizing hormone (LH), anti-Müllerian hormone (AMH) and COS treatment regimen were closely related to the GSD (*P* < 0.05). (2) The selected model has good modeling performance in terms of both root mean square error (RMSE) (29.87 ~ 34.21) and regression coefficient R (0.947 ~ 0.953). (3) A comprehensive evaluation of influencing factors for GSD is conducted and shows that the top four most significant factors are age, AMH, AFC, and BMI. (4) The proposed GSD can approximate the actual value well in the clinical application, with the mean absolute error of only 11.26 units, and the recommended results can prompt the number of oocytes retrieved (NOR) close to the optimal number.

**Conclusion:**

Modeling the GSD value with machine learning approaches is feasible and effective, and the proposed model has good clinical application for determining the GSD in the IVF treatment cycle.

## Background

The outcome of in vitro fertilization (IVF) is not only related to in vitro conditions in laboratory and personnel operations [1–3] but also closely related to the condition of oocytes. Controlled ovarian stimulation (COS) is one of the essential procedures in the IVF treatment cycle that determines the quality and quantity of oocytes. The multiple follicular growth in COS is the direct effect of exogenous Gn, which leads to supranormal circulating concentrations and recruitment of follicles whose follicle stimulating hormone (FSH) sensitivity threshold is exceeded [4, 5]. In general, the more exogenous doses of Gn, the more oocytes will be retrieved in the COS process. However, in actual clinical practice, the ovary response to Gn is complicated and can not be simply characterized by a linear relationship. Besides, growing evidence shows that an optimal number rather than a maximal oocyte retrieved is the preferred outcome of the COS process [6]. With the same dose of Gn, a poor ovarian response will lead to low quality or quantity of oocytes, resulting in a high cycle cancellation rate and low pregnancy rate [7]. In contrast, a hyper-ovarian response may contribute to high hormone levels with life-threatening side effects, such as ovarian hyperstimulation syndrome (OHSS), which will eventually lead to a higher fresh embryo transfer cancellation rate [8].

In the repeated IVF cycles, the choice of starting Gn dose is mainly based on the response observed in previous attempts, while in the first IVF cycle, the selection of the dose is primarily based on empirical methods and refers to a woman’s basic clinical characteristics such as anti-Müllerian hormone (AMH), antral follicle count (AFC), age, body mass index (BMI), etc. [9–11]. However, this strategy lacks unified standards and is suspectable to the clinician's expertise and knowledge. Till now, there is no exact recommendation standard for the dose of Gn around the world [12]. Therefore, in the context of treatment strategies aimed at optimal oocyte retrieval, customizing the dose of Gn for patients, especially the Gn starting dose (GSD), is a challenging problem faced by clinicians in the COS process.

Nowadays, some works have been conducted on building complex models based on multiple ultrasound-derived and biochemical indexes to dictate the GSD in IVF cycles [5, 13–15]. These methods, although helpful, lack comprehensive and quantifiable evaluation of the predictive performance, limiting its application in clinical practice. With the development and the increasing integration with the medical domain, machine learning is becoming more and more popular to mine the medical experience and knowledge hidden in the clinical data so as to facilitate medical practitioners to make good and scientific clinical decisions and understand the hidden principle/theory behind the phenomenon and medical data [6, 16–21].

Based on the patient's clinical data and machine learning methods, this study aims to establish a GSD prediction model that customizes the GSD and yields a good COS outcome. Besides, a comprehensive and quantitative evaluation was conducted to determine the influence of each factor on the GSD. Eventually, the proposed model is served as a scientific auxiliary tool that helps clinicians tailor the GSD in actual clinical application.

## Materials and methods

### Study design and participants

IVF/ICSI (Intracytoplasmic sperm injection) patients who underwent the first oocyte retrieving cycle in the Reproductive Center of Renmin Hospital of Wuhan University from January 2019 to December 2020 were enrolled in our study. The primary clinical characteristics such as age, infertility cause, infertility type, infertility duration, BMI, AFC and basic FSH (bFSH), Estradiol (E_2_), Luteinizing hormone (LH), AMH, therapeutic regimen, GSD and the number of oocytes retrieved (NOR) were collected. All basic endocrine levels (LH, E_2_, AMH, and bFSH) and ultrasound index AFC were detected in our hospital. Women with two conditions are excluded: (1) chromosomal abnormalities or pulmonary tuberculosis and (2) oocyte donation cycles or natural cycles. In total, 1555 women were included in a dataset to build the GSD model.

**Fig. 1:**
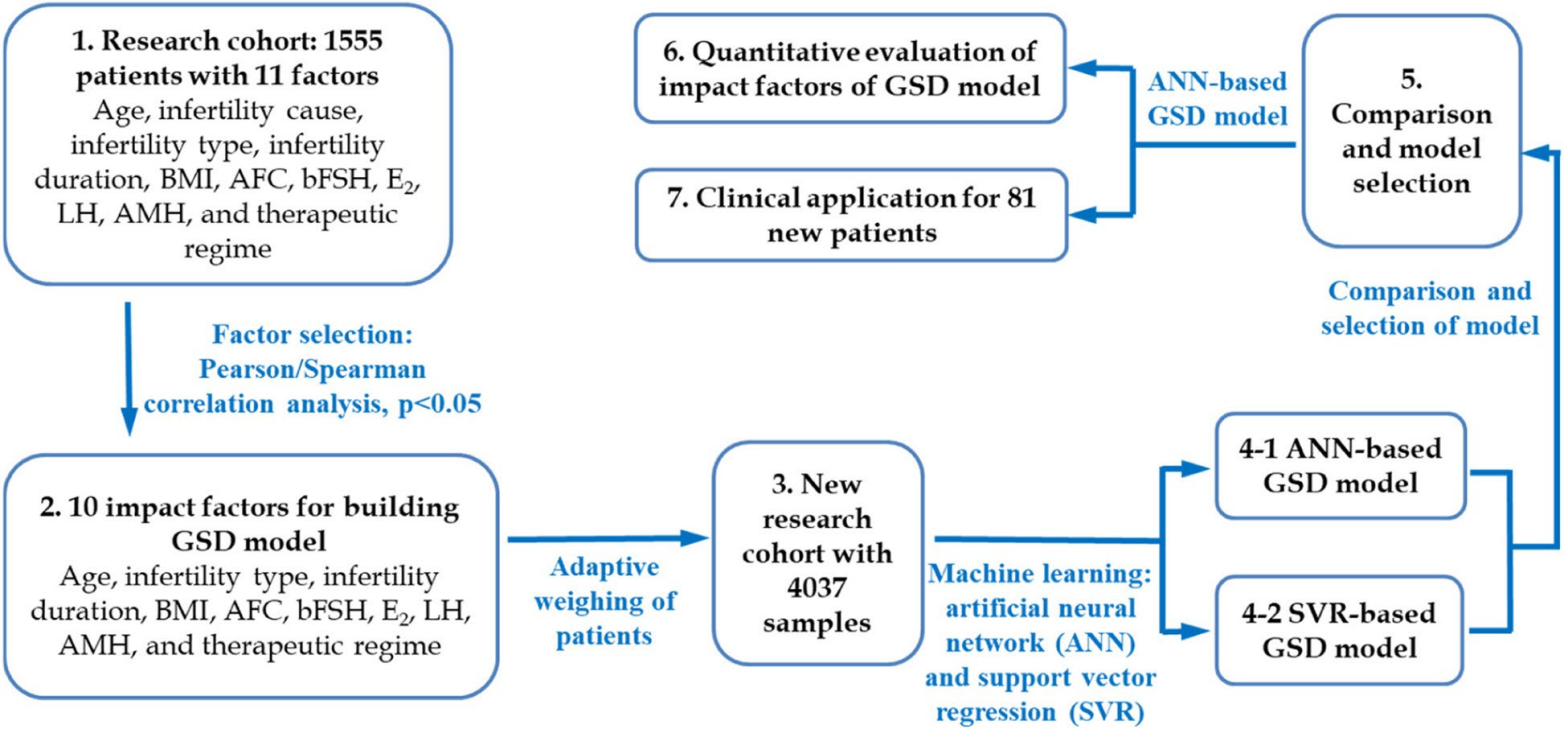
Overall framework of the proposed work. (1) Correlation analysis was conducted to find out the statistically significant factors to the GSD.(2)—(5) Incorporate the selected factors into machine learning to build the GSD model: The weight of each sample is adjusted according to the NOR, and a new research cohort that reflects the weight of sample is constructed; Based on machine learning method, the ANN-based GSD model and SVR-based GSD model are built; The prediction error and regression coefficient of the two models are compared, and the one, i.e., the ANN-based GSD model, that has better modeling performance is selected. (6) Based on the prediction results of the GSD model, the influencing factor is quantitatively and accurately evaluated. (7) 81 new patients were involved in the clinical application to verify the effectiveness of the proposed model

The overall framework of the proposed research is shown in Fig. 1.

### Machine learning methods

Since the GSD is a factor with continuous value, it is necessary to establish a regression model for GSD. Among various machine learning methods, this research selects two classical machine learning models: the artificial neural network (ANN) model and the support vector regression (SVR) model.

#### Artificial neural network (ANN) model

ANN has a wide range of applications in modeling both classification and regression problems. By selecting appropriate ANN hyperparameters, ANN can approximate any type of nonlinear function in theory, which is suitable for GSD modeling.

For the proposed ANN model, the inputs are the selected factors that are statistically significant to GSD, while the output is the GSD. The samples in the dataset were randomly divided into a training set (70%), validation set (17%), and test set (13%), which are utilized to optimize the parameters of the ANN, adjust the hyperparameters and complexity of the model, and test the generalization ability of the trained model, respectively. In the training process of the ANN model, we choose the Levenberg–Marquardt gradient updating algorithm to optimize the parameters of the ANN; the maximum number of iterations is 1500; the minimum gradient is ${1\times 10}^{-10}$; and the minimum iteration step is ${1\times 10}^{-6}$.

#### Support vector regression (SVR) model

SVR model is another popular regression modeling method of machine learning. In this research, the input and output of the SVR model are the same as the ANN model. The dataset is divided into the training set (80% samples) and the test set (20% samples). The main parameters of SVR are set as follows. SVR kernel function: Gaussian function; optimization algorithm: ISDA algorithm; The cross fold number, gradient difference tolerance, and maximum number iterations are automatically selected and optimized with hyperparameters optimization function of the SVM algorithm.

#### Evaluation indexes of the proposed model

Two indexes measure the performance of the proposed regression model: root mean square error (RMSE) and regression coefficient R. The smaller the RMSE is and the more R approaches to 1, the better the prediction performance of the proposed model.

### Adaptive adjustment weight of a sample

The objective of the proposed model is to recommend GSD in the IVF treatment cycle to obtain good IVF outcomes. The previous study has shown that, according to statistics, the best IVF results can be obtained when the NOR in the COS process is 15 ~ 18 [6].

When the NOR of a sample is closer to the optimal value (i.e., 15 ~ 18), the possibility of the rationality and feasibility of the corresponding COS strategy (including the GSD) is higher; then, we can assign the corresponding sample with a higher priority (weight) when building the GSD model. In this way, the GSD predicted from our model could also have good rationality and feasibility, potentially urging the NOR to approach the optimal value.

Here, we designed to adjust the sample weight according to the NOR adaptively. For a sample with NOR *n*, its weight is defined as:

1 $$Q\left(n\right)=\left\{\begin{array}{c}{e}^{n\left(\mathrm{ln}\left({Q}_{max}\right)-\mathrm{ln}\left({Q}_{min}\right)\right)/{(n}_{*}-{n}_{min})+\mathrm{ln}\left({Q}_{min}\right){-n}_{min}(\mathrm{ln}\left({Q}_{max}\right)-\mathrm{ln}\left({Q}_{min}\right))/{(n}_{*}-{n}_{min})}, {n}_{min}\le n\le {n}_{*}\\ {e}^{-n\left(\mathrm{ln}\left({Q}_{max}\right)-\mathrm{ln}\left({Q}_{min}\right)\right)/{(n}_{max}-{n}_{*})+\mathrm{ln}\left({Q}_{max}\right){+n}_{*}(\mathrm{ln}\left({Q}_{max}\right)-\mathrm{ln}\left({Q}_{min}\right))/{(n}_{max}-{n}_{*})},{n}_{*}<n\le {n}_{max}\end{array}\right.$$

In Eq. (1), ${n}_{*}$ is the optimal NOR, and we choose ${n}_{*}=16$; ${n}_{min}$ and ${n}_{max}$ are respectively the minimum and the maximum value of NOR for our dataset; in this work, we have ${n}_{min}=1$, ${n}_{max}=$ 47; ${Q}_{min}$ and ${Q}_{max}$ are the maximum and minimum weights of samples, respectively, and we set ${Q}_{min}=1$, ${Q}_{max}=$ 5.

**Fig. 2:**
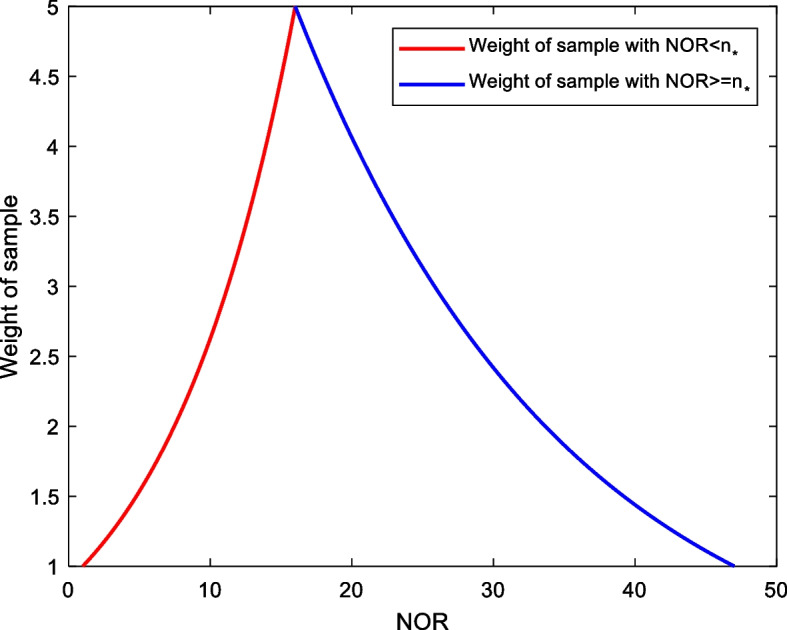
Adaptive weight of sample based on NOR

The $Q\left(n\right)$ defined in Eq. (1) is shown in Fig. 2.

As shown in Fig. 2, the $Q\left(n\right)$ of Eq. (1) is a piecewise continuous function. For a sample with NOR more approaching to ${n}_{*}$, its corresponding weight is larger, and vice versa: when ${n=n}_{*}$, ${Q\left(n\right)|}_{{n=n}_{*}}={Q}_{max}$; and when ${n=n}_{min}$ or ${n=n}_{max}$, ${Q\left(n\right)|}_{{n=n}_{min}}={Q\left(n\right)|}_{{n=n}_{max}}={Q}_{min}.$

In machine learning, directly assigning weight to a sample is complicated. In our research, this task is transferred to change the frequency of the sample in the dataset. In this way, we generate a new dataset based on the original one: for each sample in the original dataset, its frequency in the new one is set to < $Q\left(n\right)$>, where the symbol " <  > " indicates the rounding operation of a number. The new dataset generated from the original set (with 1555 samples) contains 4037 samples; and for each sample, its frequency is < $Q\left(n\right)$>. The new dataset is utilized for building the GSD model in the following part of this paper.

### Evaluation of influencing factors

To comprehensively and quantitatively evaluate the influence of each factor on the GSD, the normalized mean impact value (NMIV), as defined in our previous work [22], is calculated and analyzed. For each impact factor, e.g., the i-th factor, its NMIV (denoted by ${miv}_{i}^{*}$) is a comprehensive reflection its importance: the value of ${miv}_{i}^{*}$ represents to what extent the factor can affect the GSD; and the symbol of ${miv}_{i}^{*}$ indicates the trend of positive or negative correlation between influencing factors and the GSD: if the symbol is " + ", the larger value of the corresponding factor is, the more GSD will require, and vice versa.

### Clinical application and validation of the model

After the proposed GSD model is built based on the machine learning methods, patients who seek IVF treatment in the reproductive center of the Renmin Hospital of Wuhan University from January 2021 to February 2021 (new patients after the model is built) were selected for the clinical application and verification of the proposed model. Based on the same screening criteria in Study design and participants, we selected 81 patients for clinical application.

### Statistical and machine learning model

The data processing and correlation analysis are conducted in IBM SPSS statistics 24. Pearson correlation analysis was utilized for continuous variables, while Spearman correlation analysis was used for discretized variables in univariate correlation analysis. *P* < 0.05 was considered to be significant.

The implementation of ANN and SVR, evaluation of influencing factors, and clinical application and verification are completed in MATLAB r2021a. The prototype software for the recommendation of GSD is developed with Visual Studio 2019 and QT 6.0.

## Results

### Correlation analysis of influencing factors

The statistics of 11 influencing factors, their corresponding *P* values and the correlation coefficients are listed in Table 1. Results of correlation analysis showed that 10 factors (i.e., age, infertility type, infertility years, BMI, AFC, bFSH, E_2_, LH, AMH and therapeutic regimen) were closely related to the GSD *(P* < 0.05).

**Table 1:** Demographic and clinical characteristics of the research cohort

Influencing factor	Values	*P* value	*R*_*p*_ value
**Age** (Years)	**32.74(21–50)**	** < 0.001**	**0.301**
**Infertility type** (Number of patients)			
**Primary infertility**	**753 (48.42)**	** < 0.001**	**0.122**
**Secondary infertility**	**802 (51.57)**		
**Infertility duration** (Years)	**3.65 (0–22)**	**0.002**	**0.077**
**BMI**	**22.26 (15.0–36.2)**	**0.031**	**0.055**
**AFC** (Number of antral follicle)	**22.81 (2–65)**	** < 0.001**	**-0.357**
**bFSH** (mIU/mL)	**9.208 (0.97–151.65)**	** < 0.001**	**0.115**
**E**_**2**_ (pg/ml)	**87.19 (3.92–5086.19)**	**0.026**	**0.057**
**LH** (mIU/mL)	**4.59 (0.09–63.91)**	** < 0.001**	**-0.153**
**AMH** (ng/ml)	**3.05 (0.1–23)**	** < 0.001**	**-0.392**
Infertility cause (Number of patients)			
Pelvic and fallopian tube factors	234 (15.05)	0.224	-0.031
Polycystic ovary syndrome (PCOS) Ovulatory obstacle	108 (6.95)		
Decreased ovarian reserve	200 (12.86)		
Endometriosis	63 (4.05)		
Multiple factors	499 (32.09)		
Others	451 (29.00)		
**Therapeutic regimen** (Number of patients)			
**Long protocol**	**241 (15.50)**	** < 0.001**	**0.282**
**Super-long protocol**	**405 (26.05)**		
**Antagonist regimen**	**392 (25.21)**		
**Progestin-primed ovarian stimulation (PPOS) protocol**	**370 (23.79)**		
**Others**	**147 (9.45)**		
Gn starting dose (IU)	200.40 (5–600)	/	/
NOR	10.81 (1–47)	/	/

Values are represented as the number of women (%) or average (range)

The bold type indicates the significant influencing factors with *P* < 0.05

We will take these factors as the input to build the prediction model of GSD.

### Prediction model of GSD

Based on the results of correlation analysis, the input and output of the proposed model are:

- *Input*: age, infertility type, infertility years, BMI, AFC, basic FSH, E_2_, LH, AMH and therapeutic regimen.
- *Output*: GSD.

#### ANN-based GSD model

Among the 4037 samples in the new dataset, the number of samples in the training set, validation set, and test set are 2826 (70%), 686 (17%), and 525 (13%), respectively.

**Fig. 3:**
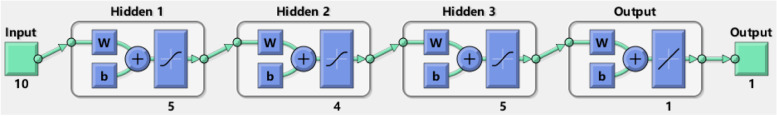
The structure of the ANN-based GSD model

In addition to the 10 inputs and 1 output, there are nodes in the hidden layers between them, which are determined by trial and error. The proposed ANN-based GSD model in this research has 3 hidden layers, including 5, 4, and 5 nodes, respectively. The structure of the proposed ANN model is shown in Fig. 3.

**Fig. 4:**
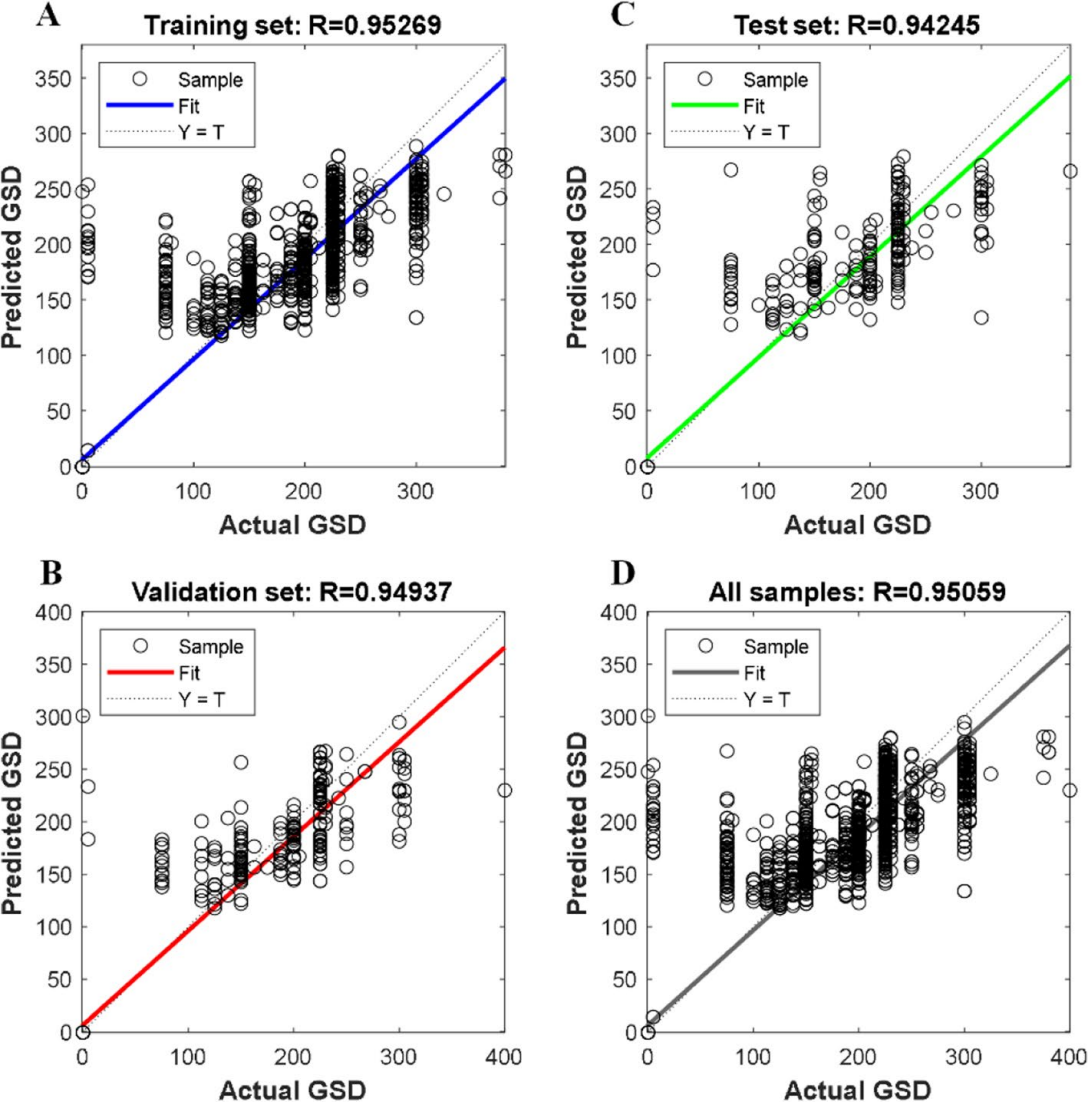
Modeling performance of ANN-based GSD model. **A** Training set; **B** Validation set; **C** Test set; **D** All samples. The horizontal and vertical axes represent the actual GSD and the predicted GSD from the model, which are denoted by the symbols Y and T, respectively

The modeling performance of the GSD-based ANN model is shown in Fig. 4 and listed in Table 2. For the training set, validation set, and test set, their RMSEs are 29.87, 32.66 and 34.21, with the regression coefficient R being 0.953, 0.949, and 0.942, respectively. For all the samples in the dataset, the RMSE and R are 31.45 and 0.951, respectively.

**Table 2:** RMSE and R of the ANN-based GSD model

ANN-based GSD model	Training set	Validation set	Test set	All samples
**RMSE**	29.87	32.66	34.21	31.45
**R**	0.953	0.949	0.942	0.951

**Fig. 5:**
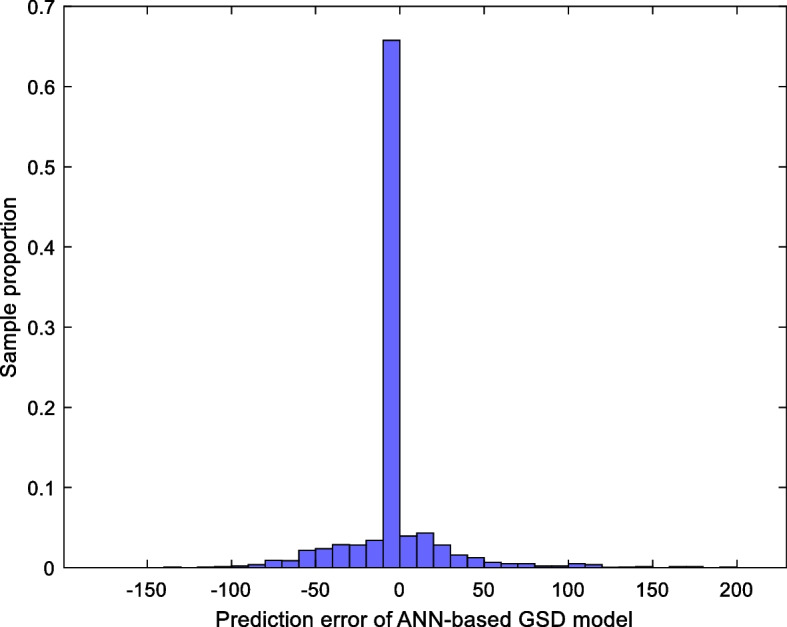
Histogram—the prediction error of the ANN-based GSD model

The histogram of the prediction error of the ANN-based GSD model for all samples is shown in Fig. 5: 77.49% of the samples have the prediction error on GSD within ± 30 units, and 87.43% of the samples have the prediction error within ± 60 units. In general, the ANN-based GSD model has good modeling performance.

#### SVR-based GSD model

For the dataset, the number of samples in the training and test sets are 3230 (80%) and 807 (20%), respectively.

**Fig. 6:**
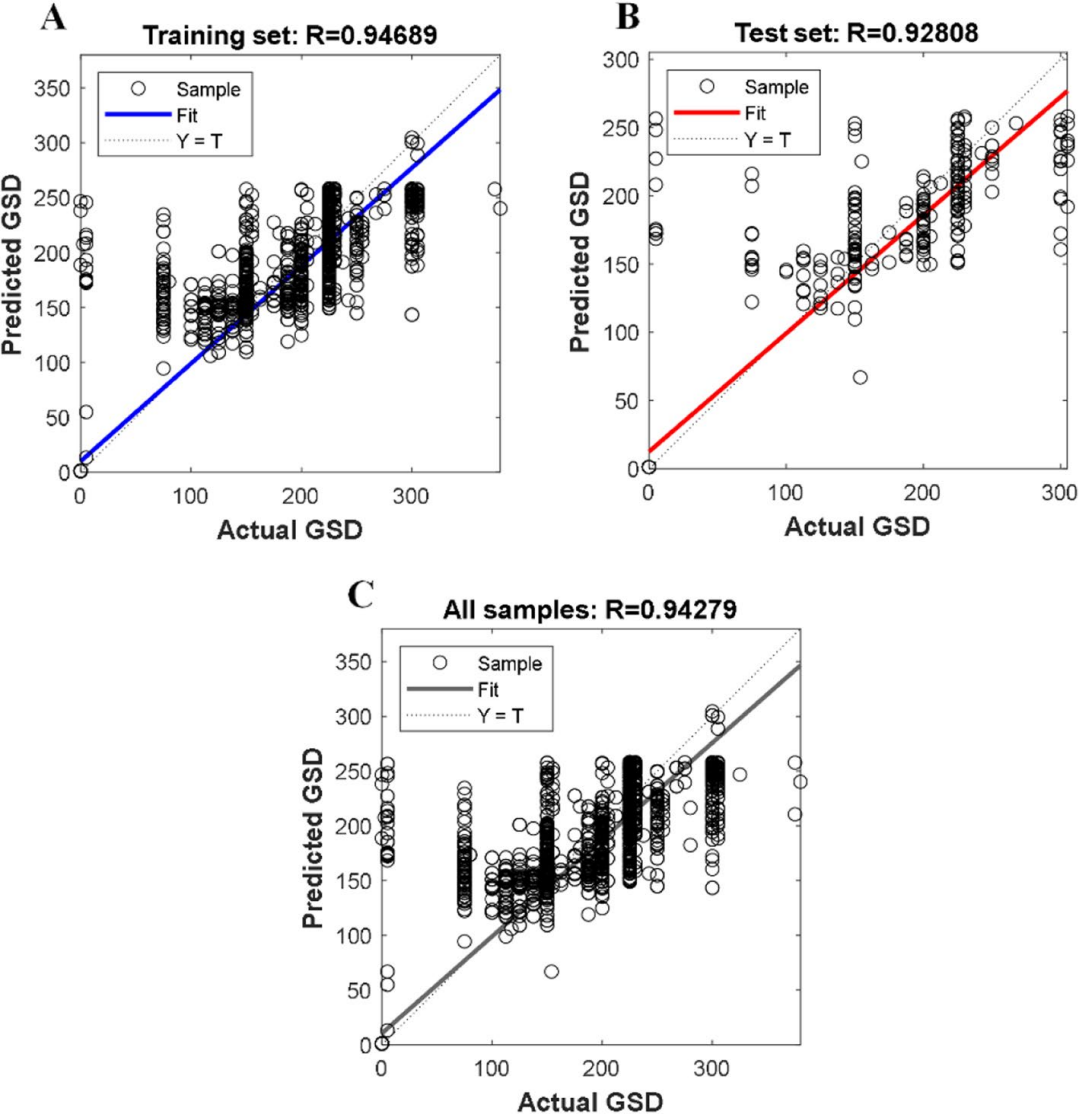
Modeling performance of SVR-based GSD model. **A** Training set; **B** Validation set; **C** All samples. The horizontal and vertical axes represent the actual GSD and the predicted GSD from the model, which are denoted by the symbols Y and T, respectively

The modeling performance of the SVR-based GSD model is shown in Fig. 6 and listed in Table 3. The RMSEs of the training and test set are 34.15 and 37.27, respectively, with their regression coefficient R being 0.947 and 0.928, respectively. For all the samples in the dataset, the RMSE and R are 34.76 and 0.943, respectively.

**Table 3:** RMSE and R of the SVR-based GSD model

SVR-based GSD model	Training set	Testing set	All samples
**RMSE**	34.15	37.27	34.76
**R**	0.947	0.928	0.943

**Fig. 7:**
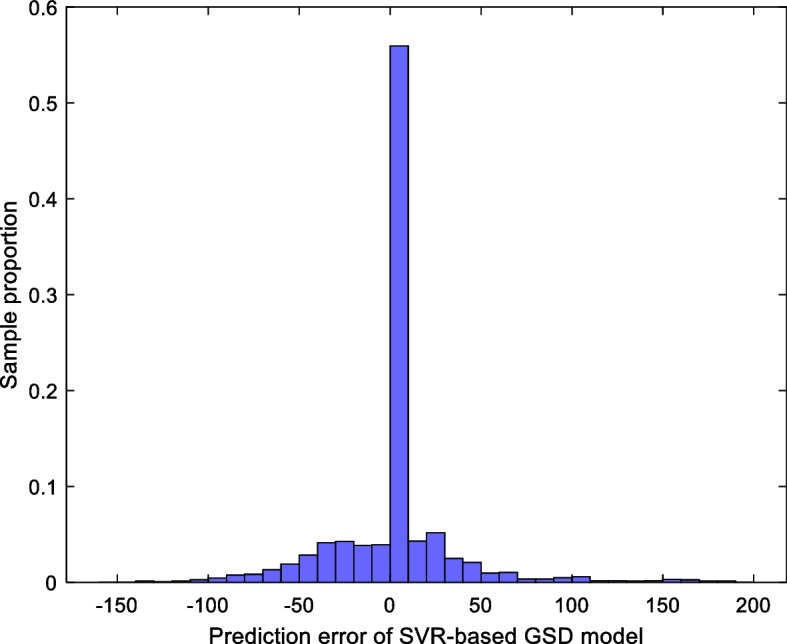
Histogram—the prediction error of the SVR-based GSD model

For the SVR-based GSD model, the histogram of its prediction error for all samples is shown in Fig. 7. Among them, 68.01% of the samples have a modeling error within ± 30 units, while 83.64% of samples have a modeling error within ± 60 units. Clearly, the SVR-based GSD model also has good prediction performance.

#### Comparision and model selection

The comparison between the above ANN model and the SVR model is listed in Table 4, which is conducted for the training set, test set, and all samples, with the RMSE being 29.87 vs. 34.15, 34.21 vs. 37.27, and 31.45 vs. 34.76, and the R being 0.953 vs. 0.947, 0.942 vs. 0.928, and 0.951 vs. 0.943. Comparison results show that the ANN performs significantly better than the SVR model. Therefore, we choose the ANN model as the final model to predict the GSD.

**Table 4:** Comparison of modeling results between ANN model and SVR model

Performance index	Model	Training set	Testing set	All samples
**RMSE**	ANN model	29.87	34.21	31.45
	SVR model	34.15	37.27	34.76
***R*** **value**	ANN model	0.953	0.942	0.951
	SVR model	0.947	0.928	0.943

### Comprehensive and quantitative evaluation of influencing factors

**Fig. 8:**
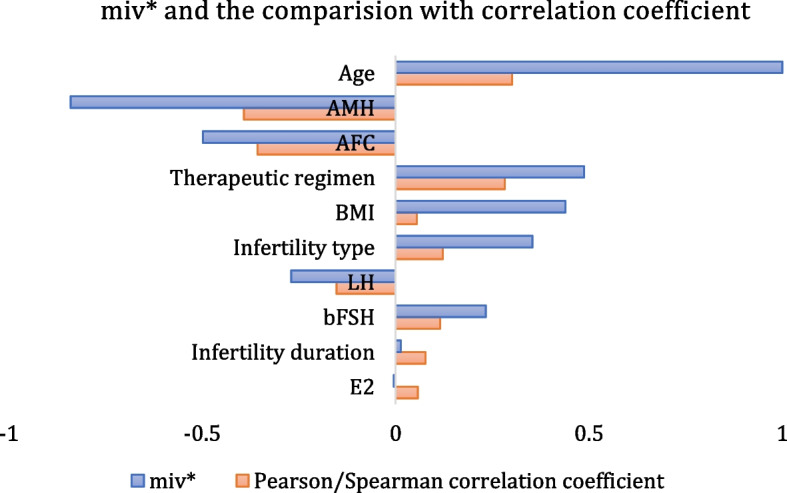
ANN-based GSD model: ${miv}^{*}$ and Pearson correlation coefficient

Based on the ANN-based GSD model and the NMIV index proposed in our previous work [22], the ${miv}^{*}$ value of each influencing factor is calculated, as listed in Table 5 and shown in Fig. 8.

**Table 5:** Normalized mean impact value (NMIV) of influencing factors

Influencing factor	Age	Infertility type	Infertility duration	BMI	AFC	bFSH	E_2_	LH	AMH	Therapeutic regimen
${miv}^{*}$	1	0.354	0.014	0.439	-0.499	0.233	-0.004	-0.270	-0.841	0.487

We can see from Table 5 that the most significant factor affecting the GSD is age (${miv}^{*}$= 1), followed by AMH (${miv}^{*}$ =—0.841), AFC (${miv}^{*}$ =—0.499), BMI (${miv}^{*}$ = 0.439), LH (${miv}^{*}$ =—0.270) and bFSH (${miv}^{*}$ = 0.233). The factor E_2_(${miv}^{*}$=-0.004)has a very small influence on GSD. Regarding the symbol of ${miv}^{*}$, the age, BMI, bFSH, and infertility duration is positive, indicating that with increasing the value of the above factors, the required GSD will increase accordingly. On the contrary, since the sign of ${miv}^{*}$ of AMH, AFC, LH, and E_2_ are negative, i.e., the greater their value, the less GSD is required for the patient.

Since the infertility type and therapeutic regimen are discretized influencing factors, their ${miv}^{*}$ are difficult to reflect the importance and trend on the GSD, which is the limitation of the index ${miv}^{*}$ [22].

### Clinical application and validation of the model

**Fig. 9:**
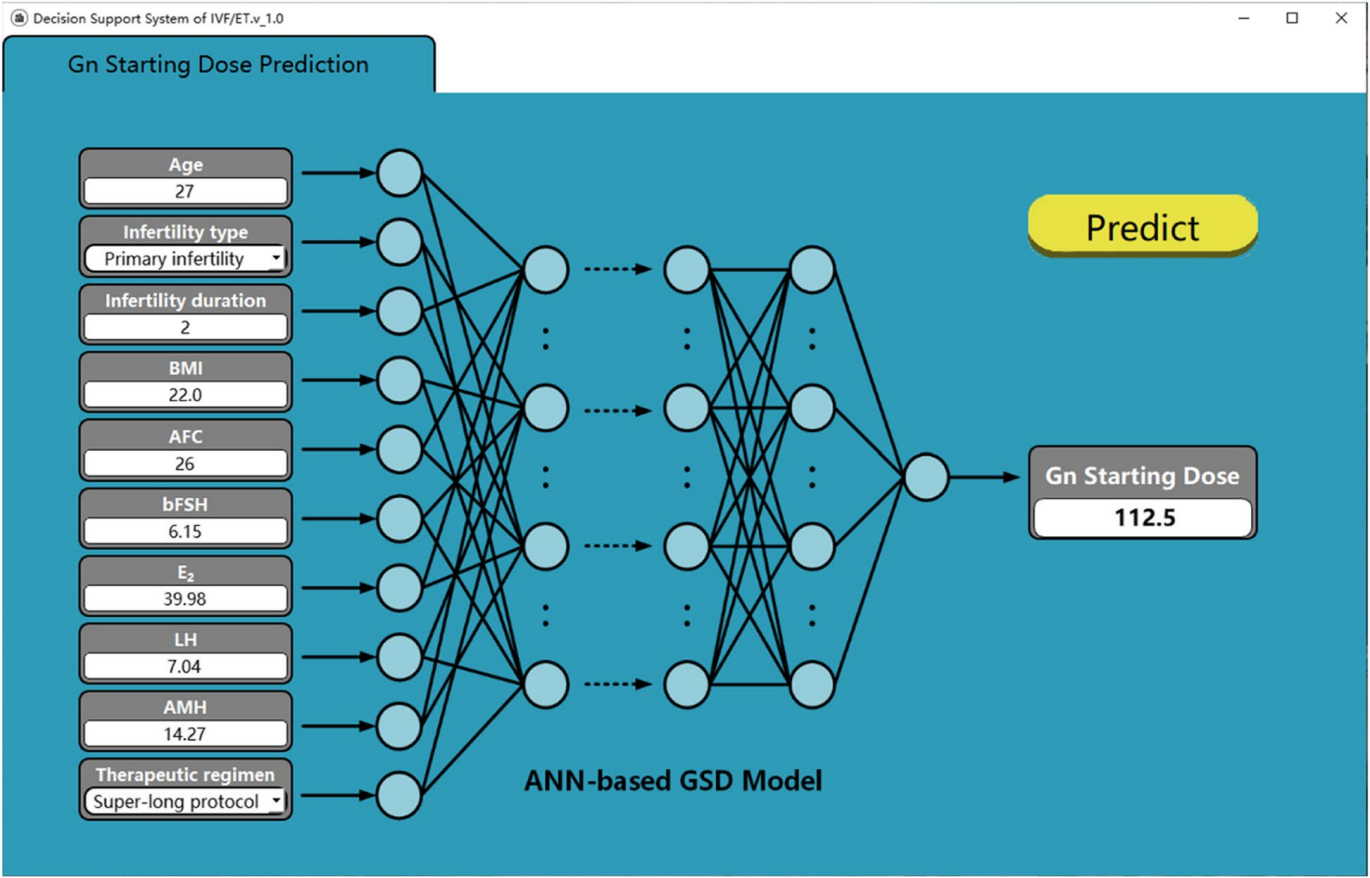
Prototype software—Decision Support System of IVF/ET – Gn Starting Dose. For each patient, the user manually inputs the values of 10 influencing factors on the left side of the software and clicks the "Predict" button on the right to call the ANN-based GSD model embedded in the software. The predicted result will show in the dialog box of “Gn Starting Dose”. The information displayed on the software interface in Fig. 9 is the clinical information of patient No. 1 and its corresponding predicted GSD

Based on the proposed ANN-based GSD model, a prototype software called “Decision Support System of IVF/ET – Gn Starting Dose Prediction” is developed, with the user interface shown in Fig. 9.

The clinical application is carried out for another 81 patients seeking IVF treatment after the ANN-based GSD model has been constructed. For each patient, the values of the 10 influencing factors (age, infertility type, infertility years, BMI, AFC, basic FSH, E_2_, LH, AMH, and treatment scheme) are input into the software, and the recommended value of GSD is calculated based on the proposed model.

**Fig. 10:**
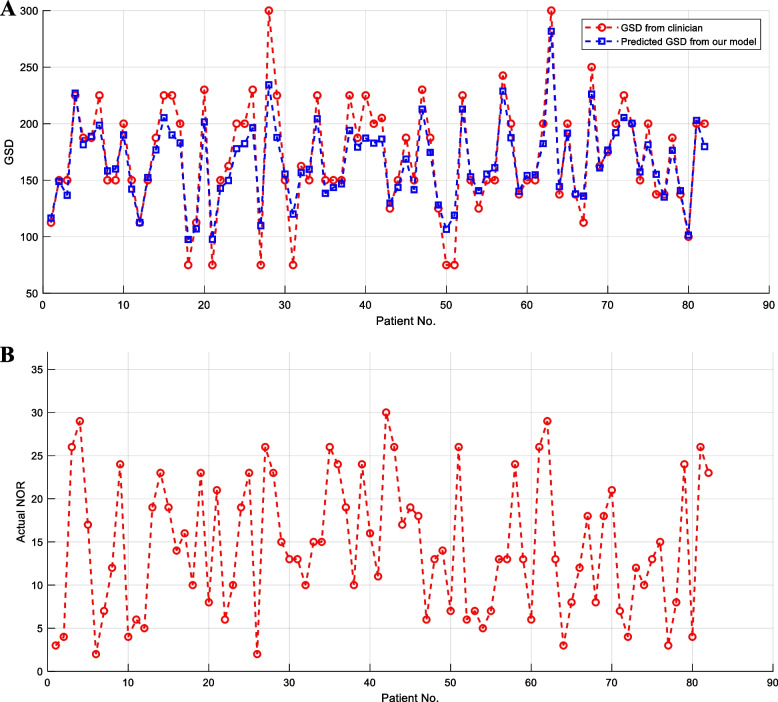
**A** Comparison between the predicted GSD from the model and the one from clinicians. The horizontal axis represents patient ID (patient No. 1 ~ 81), and the vertical axis is the GSD. **B** Actual NOR of the 81 patients. The horizontal axis represents patient ID (patient No. 1 ~ 81), and the vertical axis is the actual NOR

The GSDs of all 81 patients are calculated from the above procedure, with the results shown in Fig. 10(A). Compared with the benchmark, i.e., the GSD purely determined based on the clinician's experience and knowledge, the predicted outcome from the model can approximate it very well, with an average absolute error of only 14.08 units.

**Fig. 11:**
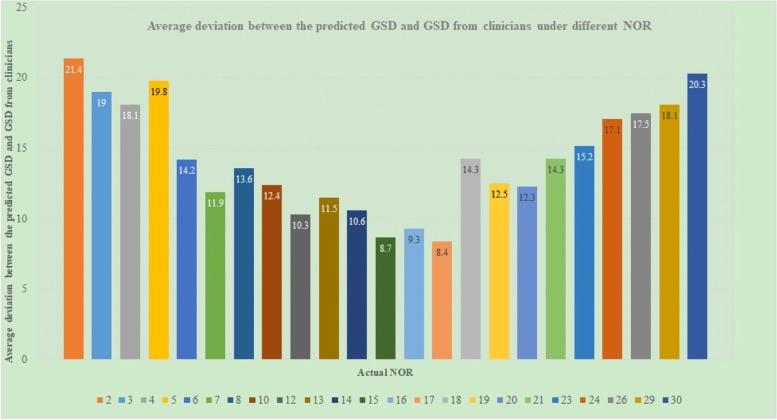
Average deviation between the predicted GSD and GSD from clinicians under different NOR

For the 81 patients, their actual NOR results are shown in Fig. 10(B). Figure 11 shows the average deviation between the predicted GSD and the GSD from clinicians under different NOR. It shows that the closer the NOR is to 16, the smaller the average deviation is. For example, the average deviation of patients with NOR = 15, 16 or 17 were 8.71, 9.33 and 8.42 units, respectively, which is significantly less than the average deviation of NOR = 2 ~ 14 oocytes or NOR = 18 ~ 30.

## Discussion

This study built a regression GSD model that can accurately predict the GSD value for the first time. Based on the prediction results of the model, a comprehensive and quantitative evaluation of the influencing factors is conducted. Compared with the traditional method that purely relies on the clinician's experience, the proposed GSD model in this study is an effective auxiliary decision-making tool for customizing the GSD in an IVF treatment cycle, as validated by both historical clinical data and real clinical applications.

Regarding tailoring the GSD in an IVF treatment cycle, some research has been done based on the indicators related to the ovarian reserve function. A prospective study of 145 "standard" patients conducted by Poporic-Todororic et al. [13] found that AFC and ovarian interstitial blood flow assessed by total power Doppler score could serve as guidance for the determination of the GSD. However, ovarian interstitial blood flow is difficult to detect in clinical practice. Marca et al. [5] proposed a GSD calculation model based on some ovarian reserve markers (age, AMH, and bFSH), which can only give rough suggestions on the GSD. Based on age, BMI, bFSH, AFC, and other indicators, Olivennes et al. [14] recently proposed the “CONSORT” algorithm to calculate the GSD. However, it has limited clinical application because it can not reduce the incidence of OHSS and the potential IVF cycle cancellation [14, 23]. Recently, Zhu et al. [15] established a GSD calculation model based on the most common ovarian reserve biomarkers. Still, it is challenging to apply to patients with a high or low ovarian response. To sum up, these studies have the problem of limited categories of biomarkers, and they lack a comprehensive and quantifiable standard for the influence factors on GSD, therefore, limiting the above methods' application in clinical practice.

Based on two machine learning methods of ANN and SVR, this study constructed two regression models for predicting the GSD. Compared with the SVR model, the ANN model has much better modeling performance. For a patient, the GSD is essentially a highly nonlinear function of its influencing factors, which is affected by continuous variables (such as age, AFC, AMH, bFSH, E_2_, LH, and so on) as well as discretized ones (e.g., infertility type and therapeutic regimen). Since the ANN method can approach any type of nonlinear model in theory and can deal with different kinds of inputs (both continuous and discretized), the ANN-based model's modeling performance is supposed to be better than that of the SVR-based model. The ANN-based GSD model constructed in this study has good prediction performance: the regression coefficient of all samples is R = 0.951, and 77.49% of the modeling error of samples is within ± 30 units. Therefore, the ANN-based GSD model is chosen and utilized in the COS process for tailoring the GSD in the IVF treatment cycle.

Based on the proposed GSD model, a comprehensive and quantitative evaluation of the influencing factors is conducted. For the GSD, the top four significant factors are: age (${miv}^{*}$=1), AMH (${miv}^{*}$=-0.841), AFC (${miv}^{*}=$-0.499) and BMI (${miv}^{*}$=0.44). That is, the older the patient and the higher the BMI value, the greater the GSD required in the IVF treatment cycle; and the higher the values of AMH and AFC, the lower GSD required. Among these factors, age is the most important one affecting the GSD. In the existing research, the importance of these factors has also been confirmed. The work [24] validates that age is the most critical factor affecting ovarian reserve function and an independent predictor of ovarian reactivity. With the increase of age, the ovarian reserve capacity becomes poor, the number and quality of follicles and responsiveness of the ovary to drugs are reduced [25, 26], and the GSD required should increase accordingly. Regarding the AMH and AFC, they can not only reflect the number of follicle recruitment but also predict low ovarian response and high response [4, 5, 27, 28]. AMH is widely used in the clinic because it is not affected by the state of the hypothalamus and oral contraceptives [29, 30]. AFC can directly reflect the reserve level of follicles, and the low-level AFC is related to low ovarian response and high pregnancy failure [28, 31]. However, due to potential error in ultrasound measurement, the accuracy of predicting ovarian reserve via AFC is limited. Therefore, when evaluating the ovarian function and tailoring the GSD, AFC is suggested to work together with other factors, such as AMH, age, etc. This also confirms our evaluation result that AMH substantially influences GSD more than AFC. What’s more, the higher the BMI, the more the GSD requires, and the proportion of OHSS complications in light-weight women is higher even using a very low GSD due to the influence of the pharmacokinetic process (drug clearance and drug dose distribution) [11, 32–34].

Regarding the therapeutic regimen, clinicians normally formulate the controlled ovarian stimulation therapeutic regimen according to their own clinical experience by considering factors like age, AFC, AMH, economic situation and complications. At present, the GnRH agonist long regimen is the most commonly used COS regiment in IVF/ICSI treatment in patients with normal ovarian response [35]. The GnRH antagonist regimen is best used for known or suspected high responders, including women with PCOS, as it reduces the risk of OHSS [36]. Although the therapeutic regimen is a discretized influencing factor, it has a high effect on the GSD value (shown in Table 1), i.e., the therapeutic regimen is a variable of the GSD prediction model. For each therapeutic regimen, as listed in Table 1, there could be a corresponding GSD per the prediction model.

In addition to the evaluation index ${miv}^{*}$, the Pearson/Spearman correlation coefficient *R*_*p*_ is calculated that reflects the univariate importance of an influencing factor on GSD, as listed in Table 1. The comparison between the ${miv}^{*}$ and *R*_*p*_ value is shown in Fig. 8. From the comparison, we know that the trend (symbol) of ${miv}^{*}$ is the same as that of *R*_*p*_ (except for E_2_). For both ${miv}^{*}$ and *R*_*p*_, the top three factors are age, AMH and AFC; however, their ranking are different. The descending ranking of *R*_*p*_ is AMH (*R*_*p*_ = -0.392), AFC (*R*_*p*_ = -0.357) and age (*R*_*p*_ = 0.301), while that of ${miv}^{*}$ value is age (${miv}^{*}$ = 1), AMH (${miv}^{*}$ = -0.841), and AFC (${miv}^{*}$ = -0.499). Since the proposed GSD model is a regression model, the ${miv}^{*}$ value calculated based on it can evaluate the influencing factors with considering the comprehensive influencing factors simultaneously, which have more scientific merit than Pearson/Spearmen coefficient *R*_*p*_ that is achieved by conducting the univariate analysis between each influencing factor and the GSD. Therefore, the comprehensive evaluation conducted in this work has better scientific merit. The existing work also partially supports our evaluation result [24].

Finally, some clinical applications of the proposed model have been conducted. Clinical application results show that our GSD model is a good mathematical expression and summary of clinicians' experience and knowledge, with the calculated results being very similar to the decision of senior clinicians (average deviation only 14.08 units). We can also see from Fig. 11 that the closer the actual NOR is to 16 (the optimal NOR in this research [6]), the smaller the average deviation could be, i.e., our GSD model can promote the NOR to be close to the optimal number of oocytes. This phenomenon follows the setting of the adaptive adjustment weight of samples in Adaptive adjustment weight of a sample. Besides, the relative average deviation of patients with NOR = 16 should be the smallest in theory; but in fact, due to the inevitable modeling error and the subjectivity and inconsistency of clinicians when deciding on GSD, the average deviation of patients with NOR = 16 is slightly higher than these with NOR = 15 and 17. In general, the closer the NOR is to 16, the smaller the average deviation could be. The results shown in Fig. 11 are indirect reflections that our model can make the NOR close to 16, and there is no direct evidence and no percentage of NOR close to 16. This is indeed a limitation of our current work.

Based on the proposed GSD model and 10 influencing factors of patients, we can get the recommended GSD in the COS process, which could serve as a good reference and basis for clinicians to tailor the GSD in the IVF treatment cycle. At the same time, the recommended GSD can make the potential NOR close to the optimal number 16.

In the future, we will investigate the effect of predicted GSD on more types of IVF-ET outcomes, like the NOR and pregnancy conditions. Also, other machine learning models considering more influencing factors will be built that could yield more types of prediction, like the outcomes of NOR and pregnancy conditions. Finally, the proposed GSD prediction model will have some further clinical applications, especially in comparing the COS result with or without the proposed GSD model.

## Conclusion

This research proposed a machine learning-based GSD model that can accurately predict the GSD in the COS process of the IVF treatment cycle. With considering the comprehensive influencing factors of GSD, the model is constructed that could precisely estimate the GSD and simultaneously could possibly make the NOR close to the optimal number. Based on our model, a comprehensive quantitative evaluation of the influencing factors is conducted. The proposed model is a good summary of the clinician’s knowledge and experience as hidden in the historical clinical data and thus can assist the clinicians in customizing GSD in a scientific and impersonal way.

## Data Availability

The datasets used and/or analysed during the current study available from the corresponding author on reasonable request.

## Abbreviations

IVF: In vitro fertilization
COS: Controlled ovarian stimulation
FSH: Follicle stimulating hormone
OHSS: Ovarian hyperstimulation syndrome
AMH: Anti-Müllerian hormone
AFC: Antral follicle count
BMI: Body mass index
GSD: Gn starting dose
ICSI: Intracytoplasmic sperm injection
bFSH: Basal follicle stimulating hormone
E_2_: Estradiol
LH: Luteinizing hormone
NOR: Number of oocytes retrieved
ANN: Artificial neural network
SVM: Supporting vector machine
RMSE: Root mean square error
NMIV: Normalized mean impact value
PCOS: Polycystic ovary syndrome
PPOS: Progestin-primed ovarian stimulation

## References

[1] Van Montfoort AP, Arts EG, Wijnandts L, Sluijmer A, Pelinck M-J, Land JA, Van Echten-Arends J (2020). Reduced oxygen concentration during human IVF culture improves embryo utilization and cumulative pregnancy rates per cycle. Human Reprod Open.

[2] Lehner A, Kaszas Z, Murber A, Rigo J, Urbancsek J, Fancsovits P (2017). Embryo density may affect embryo quality during in vitro culture in a microwell group culture dish. Arch Gynecol Obstet.

[3] Liu L, Liang H, Yang J, Shen F, Li W (2022). Analyzing the detrimental effects of female chronic hepatitis B virus DNA on ovarian reserve function and results of in vitro fertilization. Clin Exp Obstet Gynecol.

[4] Fleming R, Deshpande N, Traynor I, Yates RW (2006). Dynamics of FSH-induced follicular growth in subfertile women: relationship with age, insulin resistance, oocyte yield and anti-Mullerian hormone. Human Reprod.

[5] La Marca A, Papaleo E, Grisendi V, Argento C, Giulini S, Volpe A (2012). Development of a nomogram based on markers of ovarian reserve for the individualisation of the follicle-stimulating hormone starting dose in in vitro fertilisation cycles. BJOG.

[6] Revelli A, Gennarelli G, Biasoni V, Chiadò A, Carosso A, Evangelista F, Paschero C, Filippini C, Benedetto C (2020). The ovarian sensitivity index (OSI) significantly correlates with ovarian reserve biomarkers, is more predictive of clinical pregnancy than the total number of oocytes, and is consistent in consecutive IVF cycles. J Clin Med.

[7] Rustamov O, Wilkinson J, La Marca A, Fitzgerald C, Roberts SA (2017). How much variation in oocyte yield after controlled ovarian stimulation can be explained? A multilevel modelling study. Human Reprod Open.

[8] Sunkara SK, Rittenberg V, Raine-Fenning N, Bhattacharya S, Zamora J, Coomarasamy A (2011). Association between the number of eggs and live birth in IVF treatment: an analysis of 400 135 treatment cycles. Hum Reprod.

[9] Howles C, Saunders H, Alam V, Engrand P, Panel FTGC (2006). Predictive factors and a corresponding treatment algorithm for controlled ovarian stimulation in patients treated with recombinant human follicle stimulating hormone (follitropin alfa) during assisted reproduction technology (ART) procedures. An analysis of 1378 patients. Curr Med Res Opin.

[10] La Marca A, Argento C, Sighinolfi G, Grisendi V, Carbone M, D'Ippolito G, Carducci Artenisio A, Stabile G, Volpe A (2012). Possibilities and limits of ovarian reserve testing in ART. Curr Pharm Biotechnol.

[11] Leijdekkers JA, van Tilborg TC, Torrance HL, Oudshoorn SC, Brinkhuis EA, Koks CAM, Lambalk CB, de Bruin JP, Fleischer K, Mochtar MH (2019). Do female age and body weight modify the effect of individualized FSH dosing in IVF/ICSI treatment? A secondary analysis of the OPTIMIST trial. Acta Obstet Gynecol Scand.

[12] Farquhar C, Marjoribanks J (2018). Assisted reproductive technology: an overview of Cochrane Reviews. Cochrane Database Syst Rev.

[13] Popovic-Todorovic B, Loft A, Lindhard A, Bangsbøll S, Andersson A, Andersen AN (2003). A prospective study of predictive factors of ovarian response in ‘standard’IVF/ICSI patients treated with recombinant FSH. A suggestion for a recombinant FSH dosage normogram. Hum Reprod.

[14] Olivennes F, Trew G, Borini A, Broekmans F, Arriagada P, Warne D, Howles C (2015). Randomized, controlled, open-label, non-inferiority study of the CONSORT algorithm for individualized dosing of follitropin alfa. Reprod Biomed Online.

[15] Zhu M, Wang S, Yi S, Huang X, Meng J, Chen L, Sun H, Zhou J (2019). A predictive formula for selecting individual FSH starting dose based on ovarian reserve markers in IVF/ICSI cycles. Arch Gynecol Obstet.

[16] Scheffer JB, Scheffer BB, de Carvalho RF, Rodrigues J, Grynberg M, Mendez Lozano DH (2017). Age as A Predictor of Embryo Quality Regardless of The Quantitative Ovarian Response. Int J Fertil Steril.

[17] Yan S, Jin W, Ding J, Yin T, Zhang Y, Yang J (2021). Machine-intelligence for developing a potent signature to predict ovarian response to tailor assisted reproduction technology. Aging.

[18] Yang Y, Liu B, Wu G, Yang J (2021). Exploration of the value of progesterone and progesterone/estradiol ratio on the hCG trigger day in predicting pregnancy outcomes of PCOS patients undergoing IVF/ICSI: a retrospective cohort study. Reproductive biology and endocrinology : RB&E.

[19] Vogiatzi P, Pouliakis A, Siristatidis C (2019). An artificial neural network for the prediction of assisted reproduction outcome. J Assist Reprod Genet.

[20] Louis CM, Erwin A, Handayani N, Polim AA, Boediono A, Sini I (2021). Review of computer vision application in in vitro fertilization: the application of deep learning-based computer vision technology in the world of IVF. J Assist Reprod Genet.

[21] Merican ZZ, Yusof UK, Abdullah NL. Review on embryo selection based on morphology using machine learning methods. Int J Adv Soft Comput Appl. 2021;13(2):44–59.

[22] Liu L, Shen F, Liang H, Yang Z, Yang J, Chen J. Machine learning-based modeling of ovarian response and the quantitative evaluation of comprehensive impact features. Diagnostics (Basel). 2022;12(2):492–504. 10.3390/diagnostics12020492 PMC8871024 35204580

[23] Pouly JL, Olivennes F, Massin N, Celle M, Caizergues N, Contard F, Group FCS (2015). Usability and utility of the CONSORT calculator for FSH starting doses: a prospective observational study. Reprod Biomed Online.

[24] Liu S, Shi J (2016). Relationship between ovarian reserve & response and women age. J Reprod Med.

[25] Richardson SJ, Senikas V, Nelson JF (1987). Follicular depletion during the menopausal transition: evidence for accelerated loss and ultimate exhaustion. J Clin Endocrinol Metab.

[26] Iwase A, Nakamura T, Nakahara T, Goto M, Kikkawa F (2015). Anti-Müllerian hormone and assessment of ovarian reserve after ovarian toxic treatment: a systematic narrative review. Reprod Sci.

[27] Broer S, Dolleman M, Opmeer B, Fauser B, Mol B, Broekmans F (2011). AMH and AFC as predictors of excessive response in controlled ovarian hyperstimulation: a meta-analysis. Hum Reprod Update.

[28] McIlveen M, Skull J, Ledger W (2007). Evaluation of the utility of multiple endocrine and ultrasound measures of ovarian reserve in the prediction of cycle cancellation in a high-risk IVF population. Hum Reprod.

[29] Lan VTN, Linh NK, Tuong HM, Wong P, Howles CM (2013). Anti-Müllerian hormone versus antral follicle count for defining the starting dose of FSH. Reprod Biomed Online.

[30] Cedars MI (2022). Evaluation of Female Fertility-AMH and Ovarian Reserve Testing. J Clin Endocrinol Metab.

[31] Keane K, Cruzat VF, Wagle S, Chaudhary N, Newsholme P, Yovich J (2017). Specific ranges of anti-Mullerian hormone and antral follicle count correlate to provide a prognostic indicator for IVF outcome. Reprod Biol.

[32] Arce J-C, Andersen AN, Fernández-Sánchez M, Visnova H, Bosch E, García-Velasco JA, Barri P, De Sutter P, Klein BM, Fauser BC (2014). Ovarian response to recombinant human follicle-stimulating hormone: a randomized, antimüllerian hormone–stratified, dose–response trial in women undergoing in vitro fertilization/intracytoplasmic sperm injection. Fertil Steril.

[33] Out HJ, Rutherford A, Fleming R, Tay CC, Trew G, Ledger W, Cahill D (2004). A randomized, double-blind, multicentre clinical trial comparing starting doses of 150 and 200 IU of recombinant FSH in women treated with the GnRH antagonist ganirelix for assisted reproduction. Hum Reprod.

[34] Loy SL, Cheung YB, Fortier MV, Ong CL, Tan HH, Nadarajah S, Chan JKY, Viardot-Foucault V (2017). Age-related nomograms for antral follicle count and anti-Mullerian hormone for subfertile Chinese women in Singapore. PLoS ONE.

[35] Daya S (2000). Gonadotropin releasing hormone agonist protocols for pituitary desensitization in in vitro fertilization and gamete intrafallopian transfer cycles. Cochrane Database Syst Rev.

[36] Nardo LG, Bosch E, Lambalk CB, Gelbaya TA (2013). Controlled ovarian hyperstimulation regimens: a review of the available evidence for clinical practice. Produced on behalf of the BFS Policy and Practice Committee. Hum Fertil (Camb).

